# Effectiveness and Safety of Concurrent Use of First-Line Antiretroviral and Antituberculous Drugs in Rwanda

**DOI:** 10.1155/2014/904957

**Published:** 2014-01-30

**Authors:** Justin Ntokamunda Kadima, Marie Françoise Mukanyangezi, Claude Bernard Uwizeye

**Affiliations:** ^1^Unit of Clinical Pharmacology, Faculty of Medicine, National University of Rwanda, Huye, Rwanda; ^2^Infectious Diseases Unit, Department of Internal Medicine, Kigali University Teaching Hospital, Kigali, Rwanda; ^3^Rwanda Biomedical Centre, Kigali, Rwanda

## Abstract

*Background*. Overlapping toxicity between drugs used for HIV and TB could complicate the management of HIV/TB coinfected patients, particularly those carrying multiple opportunistic infections. This study aimed to evaluate the clinical outcomes and adverse drug events in HIV patients managed with first-line antiretroviral and first-line anti-TB drugs. *Methods*. This is a retrospective study utilizing medical dossiers from single-HIV infected and HIV/TB coinfected patients already initiated on ART. Predictors of outcomes included changes in CD4 cells/mm^3^, body weight, physical improvement, death rate, and adverse drug reactions. *Results*. Records from 60 HIV patients and 60 HIV/TB patients aged between 20 and 58 years showed that all clinical indicators of effectiveness were better in single-HIV infected than in HIV/TB coinfected patients: higher CD4 cell counts, better physical improvement, and low prevalence of adverse drug events. The most frequently prescribed regimen was TDF/3TC/EFV+RHZE. The mortality rate was 20% in HIV/TB patients compared to 8.3% in the single-HIV group. *Conclusion*. Treatment regimens applied are efficient in controlling the progression of the infection. However, attention should be paid to adjust dosing when combining nonnucleoside antiretrovirals (EFV and NVR) with anti-TB drugs to minimize the risk of death by drug intoxication.

## 1. Introduction

People living in the present century are under the threat of death as never before due to tuberculosis (TB) infection associated with human immunodeficiency virus (HIV) infection. TB is a treatable disease but remains one of the leading causes of death, particularly among people living in resource-limited countries [1, 2]. Statistics show that at least one-third of the 34 million people living with HIV worldwide are infected with latent TB, and people coinfected with TB and HIV are 21–34 times more likely to develop active TB disease than people without HIV [2–4]. Also, the rate of mortality is much greater for patients coinfected with TB and HIV. In 2011, over 700 000 people suffered from HIV associated with TB, of whom about 200 000 died, and the burden was greatest in Sub-Saharan Africa [2, 5]. As it has been proven that antiretroviral therapy (ART) reduces both case-fatality rates and the incidence of recurrent TB, the World Health Organization (WHO) recommends that ART be given to all HIV patients with extrapulmonary TB (stage 4) and to all those with pulmonary TB (stage 3), unless the CD4 count is above 350 cells/mm^3^ [6]. However, the combination of antiretrovirals (ARVs) with anti-TB drugs may precipitate intolerable adverse reactions, even death, due to the additive drug toxicities and interactions [7–9]. Therefore, initiation of ART in individuals undergoing treatment for TB, or vice versa, merits special consideration.

Since 2005, Rwanda has experienced a generalized HIV epidemic at a rate of 3% in the adult population [10]. The incidence of TB disease in Rwanda is around 361 per 100 000 population, of which 41% are coinfected with HIV [11, 12]. There are 161 TB clinics in Rwanda and all are using WHO's directly observed treatment short-course (DOTS) strategy for the management of TB disease [11]. New cases of pulmonary TB are treated with a standardized regimen of a fixed-dose combination of rifampicin, isoniazid, pyrazinamide, and ethambutol (RHZE) taken daily for 2 months (intensive phase) and then a fixed-dose combination tablet of RH administered 3 days a week for 4 months (continuation phase). As recommended by WHO, patients with TB/HIV are eligible for ART if they have a CD4+ cell count <350 cells/mm^3^.

In different health centres or hospitals providing anti-TB medications, a rise in complications or death rates has been observed among TB/HIV patients who were on cotherapy with anti-TB/ARV. These deaths or severe complications were sometimes seen within a few weeks following the initiation of ART. Currently, only a few pharmacovigilance studies have been conducted to assess the exact impact of combining ARV with anti-TB drugs on efficacy and toxicity. This study aimed at evaluating the types of combined regimens that are used, their associated effectiveness, and the occurrence of any serious side effects in both single HIV and HIV/TB coinfected patients, already on ART, when referred to a referral hospital for complications.

## 2. Materials and Methods

### 2.1. Study Site and Type

This study is a retrospective descriptive notes-based review of a series of adults with HIV or combined HIV/TB infection hospitalized in the internal medicine service at Kigali University Teaching Hospital (CHUK) in Rwanda. The CHUK with its 500-bed capacity is the largest of the four public teaching hospitals in Rwanda where some clinical trials are being conducted. In the present study, we were interested in analyzing some routine data from HIV/AIDS inpatients, where ARV and anti-TB drugs were administered, to evaluate the effectiveness and safety of the first-line regimens applied.

### 2.2. Patient Cases

Adult patients over 20 years of age and of both sexes, diagnosed with HIV-alone or HIV/TB coinfection and admitted between January 2012 and April 2013, were eligible. Patients selected were already initiated on ART at admission. They were admitted for other HIV-related or non-HIV-related infections, wasting syndrome or suspected TB. The hospital receives patients referred by other health centres upon treatment failure or development of complications. Patients had TB proven by microscopy and smear culture and HIV proven by ELISA method and western blot or PCR. According to the protocol of the hospital, patients are defined as TB-positive if at least one microbiological specimen is positive for acid-fast bacilli. Smear-negative or extrapulmonary TB is based on standard WHO case definitions [6]. The data indicated that around 86.4% and 13.6% were pulmonary and extrapulmonary TB, respectively, and the most had cryptococcus meningitis and candidiasis infection. Full blood count, liver enzymes, and serum creatinine were determined at baseline and at follow-up times. When the patients' condition deteriorated, the cause was investigated through additional diagnostic tests such as urine, stool, cerebrospinal fluid, pleural fluid and ascite analysis, blood culture, chest radiography, or abdominal ultrasound when deemed necessary by the treating physicians. New symptoms or signs were also documented as adverse events (AEs). An example of the methods used to diagnose, monitor, and manage patients during clinical trials at this hospital can be found elsewhere [13].

### 2.3. Treatment

Generally, initiation of TB treatment at CHUK requires assessment by at least two senior physicians from the department, experienced in TB care. During the period of hospitalization, all treatments were directly observed and administered by nurses in charge of care. According to the national protocol, patients with a new diagnosis of TB receive 6 months of antituberculous treatment consisting of 2 months of rifampicin, isoniazid, pyrazinamide, and ethambutol followed by 4 months of R and H (2RHZE/4RH). In the retreatment regimen 2SRHZE/1RHZE/5RHE, streptomycin (S) is added to the intensive phase. First-line ART consists of two nucleoside reverse transcriptase inhibitors (NRTIs) and a non-nucleoside reverse transcriptase inhibitor (NNRTI; nevirapine or efavirenz). National guidelines recommend initiation of ART for all extrapulmonary TB regardless of CD4 cell count and pulmonary TB with CD4 count <200 cells/mm^3^ within 2 to 8 weeks after starting antituberculous treatment. In the case of pulmonary TB with CD4 counts between 200 and 350 cells/mm^3^, initiation of ART is recommended after the intensive phase of antituberculous treatment. Preferably, all TB patients are either switched to or started on an efavirenz-based regimen because of potential drug interactions of rifampicin with nevirapine.

### 2.4. Data Collection

For this study, a data collection form was used to extract data from patient profiles, treatments received, and therapeutic outcomes. Since the study was not a clinical trial, we were only interested in extracting limited useful data for measuring treatment outcomes and side effects encountered (e.g., age, sex, type of medication, infection status, length of hospitalization, and length of ART). Indicators for antiretroviral effectiveness and safety included changes in CD4 cells/mm^3^, body weight, physical improvement, death rate, and AEs. Type and frequency of side effects experienced were used as recorded in patients' notes. Patients' files that contained many confusing data were excluded. Around 180 HIV patients were retrieved and 120 dossiers comprising 60 single HIV-alone and 60 HIV/TB coinfected cases were considered for analysis.

### 2.5. Outcome Definition

Clinical outcome was defined as improved, unimproved, or dead as indicated in the patients' notes by the treating physician. Improvement compared physical performance at the admission and exit time. Changes in weight and CD4 cell counts compared baseline values at admission and the last control values. According to the protocol in the service, an AE was defined as clinical deterioration or a new symptom occurring after the onset of treatment. The causes of AEs are commonly suspected as adverse drug reactions (ADR), concurrent infection (or neoplasm), treatment failure, paradoxical reactions, or paradoxical TB-associated immune reconstitution inflammatory syndrome (TB-IRIS). Drug-induced liver toxicity was defined as symptomatic elevation of serum transaminases (more than three times the upper limit of normal) and/or jaundice during therapy, after exclusion of other apparent causes. Renal toxicity was defined in the same way on the basis of kidney function tests.

### 2.6. Data Analysis

Data acquisition and analysis were carried out using SPSS v16 and Microsoft Excel 2007 software. Descriptive statistics were used to describe patient demographics and clinical characteristics in terms of percentages or median values. Some data were pooled to construct not more than three strata per covariate. For example, for age, we grouped patients as young (<40 years) or old (>40 years). Statistical cross-tabulation was used for data comparison using the Chi-squared test for proportions or Fisher's exact test for dichotomous variables. Significance level was set at two-sided *P* < 0.05. It was not meaningful to use Kaplan-Meier estimates to evaluate the cumulative death probability as we have only been able to control baseline values and end outcomes.

## 3. Results

### 3.1. Baseline Values

Table 1 shows the overall comparative profile of patient demographics in the two groups. The majority of patients were females (62.5% versus 37.5%). The majority of patients were less than 40 years of age 96/120 (80%). The baseline median values and range for age, weight, and CD4 cell count were 30 and 30 (20–58) years, 54 and 52 (35–65) kg, and 78 and 100 (8–280) cells/mm^3^, respectively, for the two groups.

### 3.2. Treatment Outcomes

**Figure 1:**
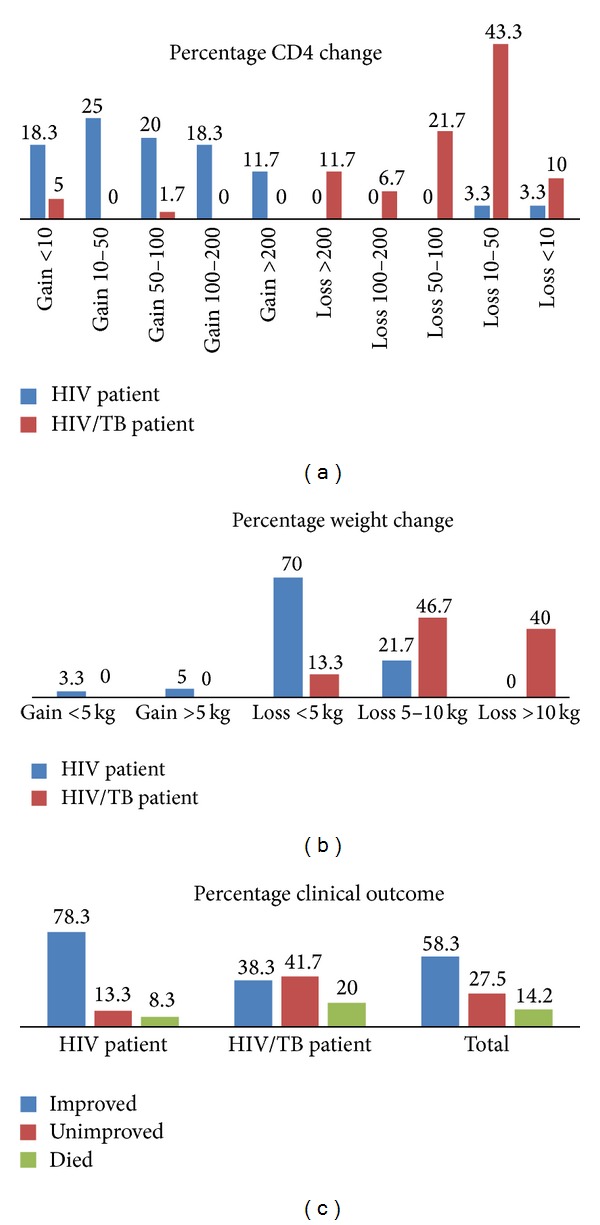
(a) Percentage weight gain or loss observed. (b) Patients' gain or loss in CD4 cells/mm^3^. (c) Physical improvement and death.

Indicators of efficacy retained in our study were body weight gain, CD4 cell count changes, physical improvement, and death. As shown in Figure 1(a), the majority of patients in both groups lost weight. The majority of the HIV-alone group had increased CD4 cell counts while the same percentage of the HIV/TB group had significantly decreased CD4 cell counts (Figure 1(b)). Seventeen out of 120 patients (14.2%) died during the period of the study (Figure 1(c)).

**Figure 2:**
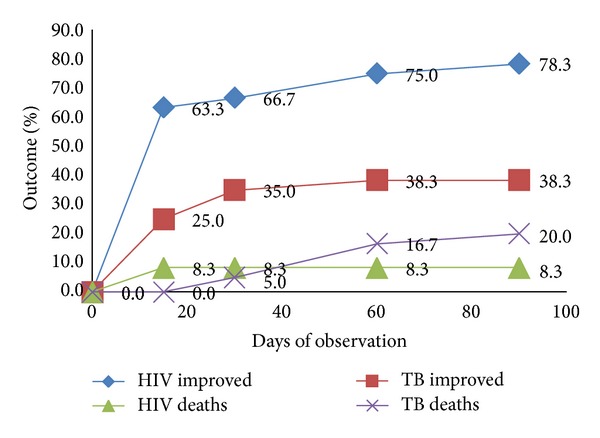
Cumulative percentages of the measured indicators in HIV-alone and TB/HIV groups. The difference is statistically significant (*P* < 0.05).

Figure 2 shows the cumulative percentage of physical improvement and deaths as observed up to 90 days in the hospital. Each patient was evaluated at the second week after admission and then one, two, and three months later during the days spent as inpatient. Every patient found to be improved medically was discharged. Patients who could not be discharged after three months were finally declared unimproved (13.3% in HIV-alone and 41.6% in TB-coinfected) while continuing treatment in the hospital.

### 3.3. Treatment Regimens and Outcomes

Table 2 presents the different drug regimens used and their related clinical outcomes. Five nucleoside reverse transcriptase inhibitors (NRTIs), lamivudine (3TC), tenofovir (TDF), abacavir (ABC), zidovudine (AZT), and stavudine (d4T), and two nonnucleoside reverse transcriptase inhibitors (NNRTIs), efavirenz (EFV) and nevirapine (NVP), were used. Three combinations were used in single-HIV patients and five in HIV/TB patients.

The most frequently used regimen was 3TC+DF+EFV in 53.3% of HIV patients and 60% of HIV/TB patients, followed by the combination 3TC+TDF+NVP in 41.7% of HIV patients and 20% of HIV/TB patients. Other combinations used ABC, AZT, and d4T. The percentages of patients judged to be improved were 80% with 3TC+TDF+NVP and 75% with 3TC+TDF+EFV. The addition of first-line anti-TB (RHZE) to these two regimens reduced the effectiveness (80% versus 33.3% for NVP and 75% versus 41.7% for EFV) while doubling the death toll (0% versus 16.7% for NVP and 15.6% versus 27.8% for EFV).

The majority of single-HIV infected patients had increased CD4 cell counts under the two backbone regimens—an 88% increase (22/25) with the 3TC+TDF+NVP regimen and a 93.8% increase (30/32) with the 3TC+TDF+EFV regimen. In the HIV/TB coinfected patients, however, the majority of patients had a decreased CD4 count—a 100% decrease (12/12) with the 3TC+TDF+NVP regimen and an 88.9% decrease (32/36) with the 3TC+TDF+EFV regimen. The difference between the two groups was statistically significant (*P* < 0.05). The number of deaths was the highest in patients under the 3TC+TDF+EFV regimen: 5/32(15.6%) in the single-HIV infected group and 10/36(27.8%) in the TB/HIV coinfected group. There were no deaths in the single-HIV group given 3TC+TDF+NVP. Regimens without TDF resulted in no mortalities, but fewer patients or none at all were judged to be improved as seen with the AZT combined regimen.

### 3.4. Other Factors Associated with Outcomes

Table 3 shows the association between some variables and patient outcomes in the two groups. We found that gender had a significant influence in HIV/TB infected patients—47.1% of females improved against 26.9% of males; 8.8% of females died compared to 34.6% of males. The most cases of death were recorded in younger patients (<40 years) regardless of the drug regimen. In 96 patients under 40 years old, there were 16 deaths while in 24 patients above 40 years of age, 2 cases of death were recorded.

### 3.5. Type and Frequency of ADEs

Table 4 depicts the types and frequencies of ADEs experienced by patients during the treatment. The most frequent complaints found were vomiting (56.7%), asthenia (45%), headache (38.3%), ulceration (36.7%), diarrhea (31.7%), and dyspnea (25%). Severe ADEs were hepatitis, kidney dysfunction, thrombocytopenia, and Stevens-Johnson syndrome. Data are reported as recorded in patients' notes. Frequencies are slightly higher in the HIV/TB group than in the HIV-alone group. In Table 4, we have added potential offensive drugs commonly reported in the literature as inducing such side effects. For example, vomiting occurred in 56.7% of all patients and may be precipitated by all ARVs or anti-TB drugs as the body's reaction to the pathophysiological condition.

## 4. Discussion

### 4.1. Demographics

TB and HIV affect all ages and both genders. In our study, we found that females outnumbered males in both single-HIV (68.3% versus 31.7%) and HIV/TB coinfected (56.7% versus 43.3%) groups and this is confirmed by other studies. In Rwanda, there is evidence that women of 20–24 years old are at much higher risk of HIV infection than their male peers [10]. According to the Rwanda Demographic and Health Survey 2010, HIV prevalence is 3.0% (3.7% for women and 2.2% for men). It has been reported that young women are more likely than men to contract HIV and most of these women are between 24 and 44 years old [14]. In Sub-Saharan Africa, women constitute 60% of people living with HIV [14, 15]. In developing countries in general, women are at an extreme disadvantage in terms of prevention and treatment of HIV, and one of the factors that put women most at risk is sexual violence [16–18]. Studies conducted in African countries have found that the first sexual experience of a girl is often forced, and during unprotected vaginal intercourse, women are more likely than men to contract HIV because HIV-infected semen has a higher viral concentration than vaginal secretions [19, 20]. Also, the gender hierarchies found within many societies contribute to the correlation of women and HIV [21]. The number of patients aged above 50 years was very small in the HIV/TB group compared to the single-HIV group (26.7% versus 3.3%). This is an indication of the impact of coinfection on the survival rate in old persons, indicating that people coinfected with HIV/TB in Africa have little chance of living over 50 years of age if infected early.

### 4.2. Treatment Regimens

The tritherapy adopted at CHUK combines two NRTIs plus one NNRTI. In the single-HIV group, the NRTIs used were TDF, 3TC, and d4T. In the HIV/TB group, AZT and ABC were added to the three used in the single-HIV group. The most frequently used combination was TDF+3TC+EFV in both groups (53.3–60%). This protocol follows WHO guidelines which recommend two NRTIs plus one NNRTI in first-line HIV regimens and RHZE as the backbone anti-TB. Worldwide, once-daily regimens comprising a nonthymidine NRTI backbone (TDF+FTC or TDF+3TC) and one NNRTI (EFV) are maintained as the preferred choices in adults, adolescents, and children older than 3 years [22, 23]. Our study showed that protease inhibitors such as lopinavir (LPV) and ritonavir (RTV) are not used at CHUK. In developed western countries, new treatments have been introduced: atazanavir (ATV), darunavir (DRV), cobicistat (COBI), elvitegravir (EVG), fosamprenavir (FPV), emtricitabine (FTC), raltegravir (RAL), and rilpivirine (RPV). The Department of Health and Human Services (DHHS) recommended regimens are EFV+TDF/FTC, ATV/RTV+TDF/FTC, and DRV/RTV+TDF/FTC [23]. The International Antiviral Society-USA (IAS-USA) recommended regimens are EFV/TDF/FTC or EFV+ABC/3TC, ATV/RTV+(TDF/FTC or ABC/3TC), and DRV/RTV+TDF/FTC [24]. These regimens are not available in Rwanda since the treatment here is low-cost or free. ARVs are delivered under NGOs or government assisted projects.

### 4.3. Treatment Effectiveness

Indicators of efficacy in our study were CD4 cell count elevation, body weight gain, physical improvement, and death rate. The majority of single-HIV patients had increased CD4 cell counts (a median of 20% over 2 months) while the same percentage of the HIV/TB group had significantly decreased CD4 cell counts (*P* < 0.05) if we compare the baseline values at entry and exit time. In the literature, it has been proved that modern ARVs can suppress viral load in people infected with HIV by up to 10 thousand times and can lead to significant increases in CD4 cell counts and this can be sustained for at least 7 years and probably indefinitely [25–27]. The slightly increased rate in HIV/TB coinfected patients is consistent with the severity of complications in this group.

The comparison of weight gain in the two groups showed that the majority of patients lost weight instead of gaining it. Normally, the expectation is to gain weight when physical health status is restored. However, treatment of HIV infection with ARVs may affect the interpretation of weight loss. Weight loss and wasting syndrome are some of the most common symptoms of HIV infection, particularly in people with certain opportunistic infections [28, 29]. HIV-infected patients treated with ART agents may lose subcutaneous fat (lipoatrophy) in the absence of fat-free mass (FFM) depletion and that can confound the clinical interpretation of weight loss [30]. Weight gain is certainly an indicator of body recovery, but it could also be related to either disease worsening or drug side effects.

The patients in our study were referred to CHUK after the development of complications and most of them had opportunistic infections treated with amphotericin, fluconazole, and co-trimoxazole. Despite the small size of the study sample examined and notwithstanding the impact of the advanced status of infection, some intriguing questions arise, such as why a higher number of deaths have been observed in patients under the TDF+3TC+EFV/RHZE regimen and why none out of six patients under 3TC+AZT+EFV+RHZE was declared improved? As indicated in Section 2, all TB patients are preferably either switched to or started on an efavirenz-based regimen because of potential drug interactions of rifampicin with nevirapine. This may mean that the worst cases would be managed with EFV combined regimen, which could explain at least in part the higher death toll. We could not observe any tentative adjusting doses. Many studies described the possible causes of death in post-HAART as AIDS related infections, non-AIDS malignancies, non-AIDS infections, liver disease, cardiovascular disease, respiratory distress, renal failure, and also drug-related side effects [31], but deep speculation is beyond the scope of the present study. Since all patients were supervised during treatment, we may exclude the impact of drug nonadherence [32].

### 4.4. Impact of Coinfection

In general, the prognosis is worse for HIV/TB patients compared to those with HIV-alone. The possible explanations may be the complications of TB disease as such, the additive side effects of combined drugs, the lack of adjustment of therapeutic dosing, for example, when efavirenz is given with rifampicin, and the poor predicted response to ART. Many studies have documented the impact of concurrent treatment with anti-TB drugs and ARVs [7–9, 43]. In our study, the great improvement of 80.0% found among HIV-alone patients compared to only 33.3% in HIV/TB patients is an indicator of complications brought about by coinfection. However, this may also indicate a previous unsuccessful response to ART. The global cure rate in Rwanda for TB and HIV separately is around 80% [11].

The death rate was higher in HIV/TB patients (12/60; 20%) than in the HIV group (5/60; 8.3%) or around two times higher. Data reported worldwide indicate that the risk of death is 1.8 times greater during cotherapy than during single anti-TB treatment [33–42]. The pattern of timing in mortality for the HIV-alone and HIV/TB groups indicated that deaths in the former group occurred soon after admission—less than 1 month (presumably through the infection precipitating admission), while those in the HIV/TB group occurred after 1 month (by slow underlying HIV and TB disease progression). Other studies showed that extensively drug-resistant tuberculosis was a cause of death in patients coinfected with tuberculosis and HIV in South Africa [3, 43]. In some cases, people have HIV that is resistant to all three of the main antiretroviral drug classes—NRTIs, NNRTIs, and PIs. There is thus room for speculating that such multiresistant cases may represent one cause of nonimprovement and death observed in our patients, without ruling out the possibility of potential drug toxicities.

### 4.5. Treatment Safety

Our patients experienced almost all the side effects frequently described with ARVs and anti-TB drugs which are vomiting, headache, ulceration, asthenia, diarrhea, dyspnea, convulsions, anemia, nightmares, skin rashes, neuropathy, renal failure, hepatitis, thrombocytopenia, Stevens-Johnson syndrome, and lipodystrophy. The rate of occurrence is likely higher in the HIV/TB group than in the HIV group. A previous study in Rwanda showed that the rate of developing a serious ADR was 35% in TB/HIV coinfected versus 7% in single-TB infected individuals [13], and this is in agreement with our findings. This confirms what has been found by others that the combination of ARVs and anti-TB drugs increases or duplicates the risk of experiencing common side effects of both those treatments [44–46]. The potential offensive drugs most reported in the literature and listed in Table 4 indicate what is likely to cause the side effects experienced. The causative relationship with each drug is beyond the scope of this study. No relationship could definitely be established between deaths and ADEs. We should accept this unless there are some unequivocal data that describe severe adverse drug reactions to specific treatments, such as TDF and renal dysfunction. This drug has been tested in a great variety of locations and not been found to be associated with severe side effects in the majority of patients [47]. Nevertheless, TDF is an acyclic nucleotide analogue reverse-transcriptase inhibitor structurally similar to the nephrotoxic drugs adefovir and cidofovir. Despite initial cell culture and clinical trial results supporting the renal safety of TDF, its clinical use is associated with a low, albeit significant, risk of kidney injury [48]. Tenofovir nephrotoxicity is characterized by proximal tubular cell dysfunction that may be associated with acute kidney injury or chronic kidney disease. Withdrawal of the drug leads to an improvement in analytical parameters that may only be partial [48].

## 5. Conclusion

The treatment regimens applied comprising first-line antiretrovirals and anti-TB, alongside aggressive antifungal therapy, are efficient in controlling the progression of the infection. However, attention should be paid when combining nonnucleoside antiretrovirals (EFV and NVR) with anti-TB drugs to minimize the risk of death by drug intoxication. This retrospective cohort study formulates hypotheses and questions to be tested in future studies and trials. The use of secondary routine data calls for some limitations.

## Figures and Tables

**Table 1:** Patients' baseline values.

Variable		HIV	HIV/TB
Gender	Male	19 (31.7%)	26 (43.3%)
	Female	41 (68.3%)	34 (56.7%)

Age (years)	<40	43 (71.7%)	53 (88.3%)
	>40	17 (28.3%)	7 (11.7%)
	Median	30 (27–46)	30 (24–33)
	IQR	21.5	9.8

Weight (kg)	<50	17 (28.3%)	22 (36.7%)
	>50	43 (71.7%)	38 (63.3%)
	Median	54 (47–58)	52 (44–58)
	IQR	12	13.5

CD4 cells/mm^3^	<200	60 (100%)	50 (83.3%)
	>200	0 (0.0%)	10 (16.7%)
	Median	78 (53–117)	100 (70–180)
	IQR	64	109.8

Median values (25th–75th percentiles).

**Table 2:** Frequency of types of regimen prescribed and their clinical outcomes.

Drug regimens		CD4 changes: *n* (within row %)		Patient outcomes: *n* (within row %)		
	Total	Gained	Lost	Improved	Unimproved	Died
	*n* (between row %)	*n* (%)	*n* (%)	*n* (%)	*n* (%)	*n* (%)
Single-HIV infected (*n*)	**60**	**55**	**5**	**47**	**8**	**5**
3TC+TDF+NVP	25 (41.7)	22 (88.0)	3 (12.0)	20 (80.0)	5 (20.0)	0
3TC+TDF+EFV	32 (53.3)	30 (93.8)	2 (6.3)	24 (75.0)	3 (9.4)	5 (15.6)
3TC+d4T+EFV	3 (5.0)	3 (100)	0	3 (100.0)	0	0
TB/HIV coinfected (*n*)	**60**	**4**	**56**	**23**	**25**	**12**
3TC+TDF+NVP+RHZE	12 (20.0)	0	12 (100)	4 (33.3)	6 (50.0)	2 (16.7)
3TC+TDF+EFV+RHZE	36 (60.0)	4 (11.1)	32 (88.9)	15 (41.7)	11 (30.5)	10 (27.8)
3TC+d4T+EFV+RHZE	2 (3.3)	0	2 (100)	2 (100)	0	0
3TC+ABC+NVP+RHZE	4 (6.7)	0	4 (100)	2 (50.0)	2 (50.0)	0
3TC+AZT+EFV+RHZE	6 (10.0)	0	6 (100)	0	6 (100)	0

Total between row percentage is calculated by dividing the frequency by 60 patients in each group. Within row percentage is calculated by dividing the frequency by the total frequency of each regimen alone.

**Table 3:** Association between various factors and clinical outcomes.

Variable	HIV-alone (*N* = 60)				HIV/TB (*N* = 60)			
	Improved	Unimproved	Died	Sign	Improved	Unimproved	Died	Sign
	*n* (%)	*n* (%)	*n* (%)	*P* value	*n* (%)	*n* (%)	*n* (%)	*P* value
Gender								
Male	17 (89.5)	0	2 (10.5)	0.116	7 (26.9)	10 (38.5)	9 (34.6)	0.037
Female	30 (73.2)	8 (19.5)	3 (7.3)		16 (47.1)	15 (44.1)	3 (8.8)	
Age (years)								
<40	36 (83.7)	2 (4.7)	5 (11.6)	0.004	21 (39.6)	22 (41.5)	10 (18.9)	0.782
>40	11 (64.7)	6 (35.3)	0		2 (28.6)	3 (42.9)	2 (28.6)	
Weight (kg)								
Gained	5 (100)	0	0	0.009	0	0	0	0.032
Lost	42 (83.3)	8 (4.8)	5 (11.9)		23 (38.3)	25 (41.7)	12 (20.0)	
CD4 (cells/mm^3^)								
Gained	42 (76.4)	8 (14.5)	5 (9.1)	0.001	3 (27.3)	5 (45.4)	3 (27.3)	0.000
Lost	4 (100)	0	0		20 (40.8)	20 (40.8)	9 (18.4)	
ART length								
<1 year	9 (75.0)	0	3 (25.0)	0.107	6 (100)	0	0	0.003
1–5 years	25 (78.2)	5 (15.6)	2 (6.2)		11 (36.7)	10 (33.3)	9 (30.0)	
>5 years	13 (81.2)	3 (18.8)	0		6 (25.0)	15 (62.5)	3 (12.5)	
Column percent	**47 (78.4)**	**8 (13.3)**	**5 (8.3)**		**23 (38.4)**	**25 (41.6)**	**12 (20)**	

Total between row percentage is calculated by dividing the frequency by 60 patients in each group. Within row percentage is calculated by dividing the frequency by the total frequency of each regimen alone. Sign: *P* < 0.05 Pearson Chi-square.

**Table 4:** Types and frequency of ADEs experienced by patients.

Adverse drug events	HIV (*N* = 60)		HIV/TB (*N* = 60)		Total (*N* = 120)		Potential offensive drugs
	*n*	%	*n*	%	*N*	%	
Vomiting	32	26.7	36	30.0	68	56.7	All ARV and anti-TB
Asthenia	22	18.3	32	26.7	54	45.0	EFV, TDF, H
Headache	26	21.7	20	16.7	46	38.3	EFV
Ulceration	24	20.0	20	16.7	44	36.7	EFV, AZT, 3TC
Diarrhea	18	15.0	20	16.7	38	31.7	Many ARV, anti-TB
Dyspnea	12	10.0	18	15.0	30	25.0	ABC, EFV, 3TC, AZT, R
Convulsions	10	8.3	12	10.0	22	18.3	EFV, H
Anemia	8	6.7	12	10.0	20	16.7	AZT, 3TC, R, E
Hepatitis	5	4.2	14	11.7	19	15.8	3TC, NVP, R, H, Z
Skin rashes	8	6.7	10	8.3	18	15.0	EFV, NVP, R, Z, E
Kidney dysfunction	6	5.0	10	8.3	16	13.3	TDF, R
Nightmares	8	6.7	6	5.0	14	11.7	EFV
Thrombocytopenia	4	3.3	6	5.0	10	8.3	AZT, 3TC, H
Neuropathy	6	5.0	3	2.5	9	7.5	d4T, H
Stevens-Johnson syndrome	4	3.3	4	3.3	8	6.7	EFV, NVR
Lipodystrophy	2	1.7	1	0.8	3	2.5	d4T

Potential offensive drugs: most reported in the literature likely to cause the side effects experienced. The causative relationship with each drug is beyond the scope of this study.
